# Predictors of intimate partner violence and associated marital disruption among ever-married women in sub-Saharan Africa: a multi-country analysis for policy and intervention priorities

**DOI:** 10.3389/fsoc.2025.1658603

**Published:** 2025-10-13

**Authors:** Judith Ifunanya Ani, Lucky Norah Katende-Kyenda

**Affiliations:** Faculty of Medicine and Health Sciences, Walter Sisulu University, Mthatha, South Africa

**Keywords:** Intimate Partner Violence (IPV), marital disruption, Demographic and Health Surveys (DHS), sub-Saharan Africa (SSA), public health

## Abstract

**Introduction:**

Intimate Partner Violence (IPV) remains a significant public health and human rights concern globally, disproportionately affecting women. This study investigates predictors of IPV and its association with marital disruption among ever-married women in sub-Saharan Africa.

**Methods:**

Cross-sectional data from the Demographic and Health Surveys (DHS) covering 25 sub-Saharan African countries was analysed. The study examined the prevalence of IPV and marital disruption, focusing on socio-demographic characteristics (age, residence, education, and union duration) and partner-related factors (controlling behavior, alcohol use, age differences, and exposure to parental violence).

**Results:**

Findings revealed a high prevalence of IPV across the study population. IPV was significantly associated with marital disruption, particularly among women aged 25–29, those residing in rural areas, with primary education, and those in unions lasting 5–9 years. Partner characteristics, including controlling behavior, alcohol consumption, larger age gaps, and a history of witnessing parental violence, were strongly linked to both IPV and increased risk of separation or divorce. Women who experienced any form of IPV had 56% lower odds of remaining in a current union, highlighting IPV as a major driver of marital instability.

**Discussion:**

These results underscore the urgent need for targeted, context-specific interventions to address IPV and mitigate its destabilizing effects on family structures. Policy recommendations include advancing women’s education and economic empowerment, promoting community-level awareness campaigns, and transforming harmful gender norms. Such interventions are critical for achieving Sustainable Development Goal 5 on gender equality and ensuring women’s safety and stability in intimate relationships.

## Introduction

One of the key objectives of the Sustainable Development Goals (SDGs) is to promote equal opportunities for all genders and reduce disparities across different sectors (United Nations, 2015a,b). However, achieving these goals continues to face significant setbacks due to the ongoing rise in gender-based violence (GBV) across various aspects of life. For instance, the World Health Organization (2021) estimates that nearly one in three women worldwide (30%) have experienced physical or sexual intimate partner violence (IPV) or non-partner sexual violence in their lifetime, with some sub-Saharan African countries reporting rates as high as 40–60% (World Health Organization, 2021). Among the various forms of GBV, domestic violence remains particularly prevalent, hindering progress toward these global goals. IPV, a widespread form of domestic violence, poses serious social, health, and human rights challenges (Ahinkorah et al., 2018; World Health Organization, 2014). It encompasses physical, emotional, or sexual harm inflicted by a current or former partner (Wagman et al., 2016). In its 1993 Declaration on the Elimination of Violence against Women, the United Nations General Assembly defined domestic violence as “any act of gender-based violence that causes or is likely to cause physical, sexual, or psychological harm or suffering to women. This includes threats of such acts, coercion, or arbitrary deprivation of liberty, whether occurring in public or private life” (Kishor, 2005).

A significant concern is the deep-rooted acceptance of IPV as a form of discipline in many African societies, perpetuating gendered power imbalances (Rydstrøm, 2010; Ajayi and Soyinka-Airewele, 2018; Ampofo and Prah, 2009; Ray and Qayum, 2009). In such cultural contexts, women—like children—are often expected to submit fully to their husbands. Even more alarming is the social stigma faced by women who report violence, as they are frequently perceived as bringing dishonor or scandal upon their families or husbands (Arisukwu et al., 2021). Consequently, many women remain silent and endure abuse, leading to numerous unreported cases and fatalities.

Various studies highlight the high occurrence of IPV in patrilineal societies characterized by male dominance (Igbolekwu et al., 2021; Bamiwuye and Odimegwu, 2014). For example, Bamiwuye and Odimegwu (2014) reported alarming rates of physical, sexual, and emotional violence across sub-Saharan Africa, ranging from 30.5% in Nigeria to 57.6% in Cameroon, with Zimbabwe (43.4%), Kenya (45.3%), Mozambique (45.5%), and Zambia (53.9%). Interestingly, domestic violence rates were higher in wealthier households than in those with lower or middle incomes (Bamiwuye and Odimegwu, 2014), indicating that IPV transcends socioeconomic status. Similarly, Seidu et al. (2021) found that women experiencing physical, sexual, or emotional violence were more likely to face marital disruptions than those who had not, underscoring how IPV not only harms women but also contributes to household instability. These findings highlight the urgent need for comprehensive interventions that address IPV and promote safer, more stable relationships in these communities. Furthermore, more recent analyses further indicate that IPV escalated during the COVID-19 pandemic, as lockdowns intensified women’s exposure to abusive partners and limited their access to support systems (Piquero et al., 2021). These findings underscore that IPV, not only as a pervasive human rights violation but also as a destabilizing factor in family and social structures, continues to hinder progress toward gender equality in the region.

IPV remains pervasive in sub-Saharan Africa. Marital disruption, including divorce and separation, reflects not only the breakdown of spousal relationships but also signals deeper psychosocial and economic consequences for women and their families. Existing research indicates that IPV erodes relationship quality, breeds mistrust and fosters conditions that precipitate marital breakdown (Seidu et al., 2021; Wagman et al., 2016). Various factors—such as education, wealth status, duration of union, partner’s alcohol use, controlling behavior, media exposure, and women’s empowerment—serve both as risk factors for IPV and predictors of relationship stability. For example, early cohabitation, witnessing parental violence, and limited economic autonomy may increase vulnerability to abuse and reduce the likelihood of leaving abusive relationships. Conversely, media exposure and higher education levels can enhance awareness and agency, potentially increasing the chances of exiting violent unions. Understanding how these variables associate with IPV and marital disruption is critical for designing interventions that prevent violence and support women navigating relationship transitions.

As a consequence of certain factors, this study focuses on IPV and marital dissolution through divorce or separation. Research across 33 African countries indicates that approximately 25% of marriages end in divorce within the first 15–19 years of union (Chisumpa and Chirwa-Banda, 2020; Clark and Brauner-Otto, 2015; Adegoke, 2010). Given the central role of family structures in society, marital dissolution has profound social, emotional, economic, and health impacts on partners and families—especially when children are involved. Previous studies demonstrate the high prevalence of IPV in sub-Saharan Africa, with severe consequences such as death and depression for women, many of which remain unreported (Gubi et al., 2020; Ani et al., 2019; Wagman et al., 2016; World Health Organization, 2013a,b). The COVID-19 lockdown further prompted re-evaluation of domestic violence patterns in the region (Piquero et al., 2021).

Despite extensive research on IPV and its consequences, significant gaps remain in understanding the relationship between the experiences of different forms of IPV and marital disruption across diverse contexts. Most existing studies focus on single countries or specific regions, limiting the generalizability of findings (Garcia-Moreno et al., 2006; Jewkes and Morrell, 2010). Furthermore, much of the literature treats IPV as a unidimensional phenomenon, with limited attention to how physical, emotional, and sexual violence distinctly influence marital outcomes (Devries et al., 2013; Capaldi et al., 2012). There is also a paucity of large-scale, multi-country analyses using representative population data—particularly within sub-Saharan Africa, where IPV prevalence and its social consequences remain critically underexplored (Garcia-Moreno et al., 2006; Jewkes and Morrell, 2010). Finally, few studies explicitly link IPV and marital disruption to broader policy frameworks such as the SDGs, missing opportunities to inform intervention priorities (Heise et al., 2019; United Nations, 2015a,b). This study seeks to fill these gaps by providing a multi-country analysis of predictors of IPV’s various forms and their association with marital disruption using Demographic and Health Survey data, with a focus on informing targeted interventions. By addressing these gaps, the present study contributes to ongoing efforts to inform gender and social inclusion policies and to guide the development of effective interventions targeting IPV and marital disruption in sub-Saharan Africa.

## Methods

### Data source and design

This study adopted a cross-sectional design using nationally representative data from the Demographic and Health Surveys (DHS). The DHS datasets used for this study were conducted between 2012 and 2022 (refer to Table 1). The DHS, conducted in over 80 countries, employs standardized sampling, questionnaire design, and data collection methods, which enables cross-country comparisons of key health and social indicators. For this analysis, we focused on ever-married women aged 15–49 who participated in both the domestic violence module and relevant demographic modules. Only the most recent survey for each country was included to ensure the data reflected current trends. Ethical clearance for each country was obtained by ICF Macro and respective national authorities. The datasets used for this research are accessible at https://dhsprogram.com/data/available-datasets.cfm.

**Table 1:** Sampled countries in SSA.

S/N	Countries	Year
1	Angola	2015–16
2	Benin	2017–18
3	Burundi	2016–17
4	Cameroon	2018
5	Chad	2014–15
6	Comoros	2012
7	Congo DR	2013–14
8	Gabon	2019–21
9	The Gambia	2019–20
10	Kenya	2022
11	Liberia	2019–20
12	Madagascar	2021
13	Malawi	2015–16
14	Mali	2018
15	Mauritania	2019–21
16	Namibia	2013
17	Nigeria	2018
18	Rwanda	2019–20
19	Sierre Leone	2019
20	South Africa	2016
21	Tanzania	2015–16
22	Togo	2013–14
23	Uganda	2016
24	Zambia	2018
25	Zimbabwe	2015

### Inclusion criteria

The DHS datasets used for this study were conducted between 2012 and 2022. The datasets contained the domestic violence module used for analysis. Women were eligible for inclusion if they had ever been in a marital or cohabiting union and completed all relevant IPV-related items.

### Study variables

#### Outcome (dependent) variable

The study examined two primary outcome variables: IPV and marital disruption. IPV was measured using three dimensions: physical, emotional, and sexual violence. These were operationalized based on responses to a series of standardized DHS questions. Women were considered to have experienced IPV if they answered “Yes” to any item under the physical, emotional, or sexual abuse domains.

To measure physical IPV, respondents were asked if their (current or most recent) partner ever hit, slapped, kicked or did anything to do them harm physically. These were the questions used to measure physical violence:

Does (did) your (last) husband/partner ever do any of the following things to you?

1. Push you, shake you, or throw something at you? Slap you?
2. Twist your arm or pull your hair?
3. Punch you with his fist or with something that could hurt you?
4. Kick you, drag you, or beat you up?
5. Try to choke you or burn you on purpose?
6. Threaten or attack you with a knife, gun, or any other weapon?

Emotional IPV included instances where a partner humiliated the woman in front of others, threatened her or someone close to her, or insulted her to make her feel bad. These questions were used to measure emotional violence:

Does (did) your (last) husband/partner ever:

1. Say or do something to humiliate you in front of others?
2. Threaten to hurt or harm you or someone close to you?
3. Insult you or make you feel bad about yourself?

Sexual IPV involved being physically forced to have sexual intercourse or perform unwanted sexual acts against the woman’s will or without her consent. These are the questions:

1. Physically force you to have sexual intercourse with him even when you did not want to?
2. Force you to perform any sexual acts you did not want to?

These were coded as a dichotomous variable (Yes/No). Women who experienced any or all of these were coded as ‘Yes,’ while those who did not experience any were coded as ‘No’.

Marital disruption was derived from the DHS marital status question. Marital categories included never in a union, married, living with a partner, widowed, divorced, separated. Women who reported being “separated” or “divorced” at the time of the survey were classified as experiencing “marital disruption.” Those who reported being “married” or “living with a partner” were considered to be in intact unions or “not disrupted.” Women who were widowed or never in a union were excluded from the analysis. In other words, respondents were classified as either having a disrupted marriage or not having a disrupted marriage. Using a binary outcome, “1” indicates that a woman is currently married or cohabiting, and “0” indicates that she is divorced or separated.

### Explanatory (independent) variables

The explanatory (independent) variables were classified into two categories: background characteristics and intermediate factors.

*Background characteristics* included socio-demographic factors and economic empowerment.

Sociodemographic factors include age, women’s education level, place of residence, regions, wealth status index, partner’s education, parity (number of children), age difference, employment status, exposure to media.

- Age included: “15–19,” “20–24,” “25–29,” “30–34,” “35–39,” “40–44,” and “45–49.”
- Women’s education level—no formal education, primary, secondary and tertiary.
- Partner’s education also included no formal education, primary, secondary and tertiary.
- Place of residence was coded as “urban” and “rural.”
- Wealth index was estimated using the DHS measure of wealth as a composite variable derived by combining certain household data including materials used to construct houses, type of access to water, facilities for sanitation and assets ownership. These were categorized into five wealth quintiles namely poorest, poorer, middle, richer and richest.
- Age difference with—wife older, 0–5-year gap and 6 years and above age gap,
- Parity was categorized as “None,” “1–4” and 5+.
- Employment status or current working status was coded as currently employed or unemployed.
- Exposure to visual and print media (Media exposure) was created and coded as “exposed” and “not exposed.” A woman was considered as “exposed” is she listened to, watched or read from the mass media that included television, radio, social media, newspaper, magazines at least once a week or almost every day. A woman was considered “not exposed” if she did not listen to, watched or read from the mass media that included television, radio, social media, newspaper, magazines at all or less than once a week. To do this, we computed the frequency as an index variable and then coded as “exposed” or” not exposed.”

Economic empowerment was defined based on female ownership of property and the nature of earnings from the respondent’s work. Property ownership was assessed by combining responses to the following questions: (a) Does the respondent own a house (either solely or jointly with a partner)? and (b) Does the respondent own land (either solely or jointly with a partner)? In addition, respondents were asked whether their work was remunerated—categorized as either not paid or paid (in cash, in kind, or both). Responses to these items were dichotomized as “Empowered” or “Not Empowered.” A woman was classified as economically “Empowered” if she answered “Yes” to owning a house, owning land, or receiving any form of payment for her work.

*Intermediate factors* included partners’ behavioral factors, history of witnessing parental violence and marital factors.

Partner’s behavioral factors were measured as partner’s controlling behaviors and partner’s alcohol consumption. Partner’s controlling behavior, measured by whether the partner prohibited the woman from seeing friends or family, monitored her movements, showed excessive jealousy, or frequently accused her of infidelity. These were captured with the questions below that asked the respondent if the partner;

i) Prohibit you to meet female friends?
ii) Limit you contact your family?
iii) Insist on knowing where you are at all times?
iv) Is jealous if you talk with other men?
v) Frequently accuses you of being unfaithful?

When a respondent answered “Yes” to any of the five questions, partners controlling behavior was implied but when it is a “No” to all the questions, it was considered absence of partner’s controlling behavior.

Partner’s alcohol consumption was measured with the question: Does your partner drink alcohol?” This was coded as “Yes” or “No.” And if ‘yes,” frequency of alcohol consumption (or frequency of intoxication) was categorized as “Never gets drunk,” “Often” and “Sometimes.”

History of violence was measured by determining witnessing parental violence, that is, whether the respondent’s father ever beat her mother. This was coded as “Yes” or” No.”

Marital factors included for the study were duration of relationship, number of co-wives, age at first cohabitation/marriage and parity. Duration of relationship was coded as 0 = 0–4 years, 1 = 5–9 years, 2 = 10–14, 3 = 15–19 and 4 = 20+ years. Number of co-wives were classified as “None” and “One or more co-wives.” Age at first cohabitation/marriage were classified as a dichotomous variable “below 18 years” and “18 years and above.”

### Data analysis

Data analysis involved multiple steps. First, the prevalence of IPV was assessed by computing frequencies and percentages at the descriptive level. Univariate analysis was employed to describe the socio-demographic characteristics of respondents. Pearson’s chi-square tests were conducted to examine associations between IPV and the explanatory variables.

Subsequently, multivariate logistic regression was performed to determine the relationship between the explanatory variables and experiences of IPV.

Further analysis involved cross-tabulation of each form of IPV with marital disruption. A composite IPV variable was constructed, and its association with marital disruption was tested using Pearson’s chi-square test of independence (χ^2^), with statistical significance set at *p* < 0.05. Finally, multilevel binary logistic regression was used to assess the predictive relationship between IPV and marital disruption. This was extended by using the composite IPV variable to examine its predictive power on marital disruption.

### Ethical considerations

Ethical approval for the study was granted by the Ethics Committee of ICF Macro International, Inc., Calverton, Maryland, as well as by the National Ethics Committees of each participating country. Although the dataset used is publicly available, formal permission to access and use the data was obtained. Comprehensive information on the dataset and adherence to ethical standards is available at: http://goo.gl/ny8T6X.

## Results

### Prevalence of IPV in sub-Saharan Africa

**Figure 1:**
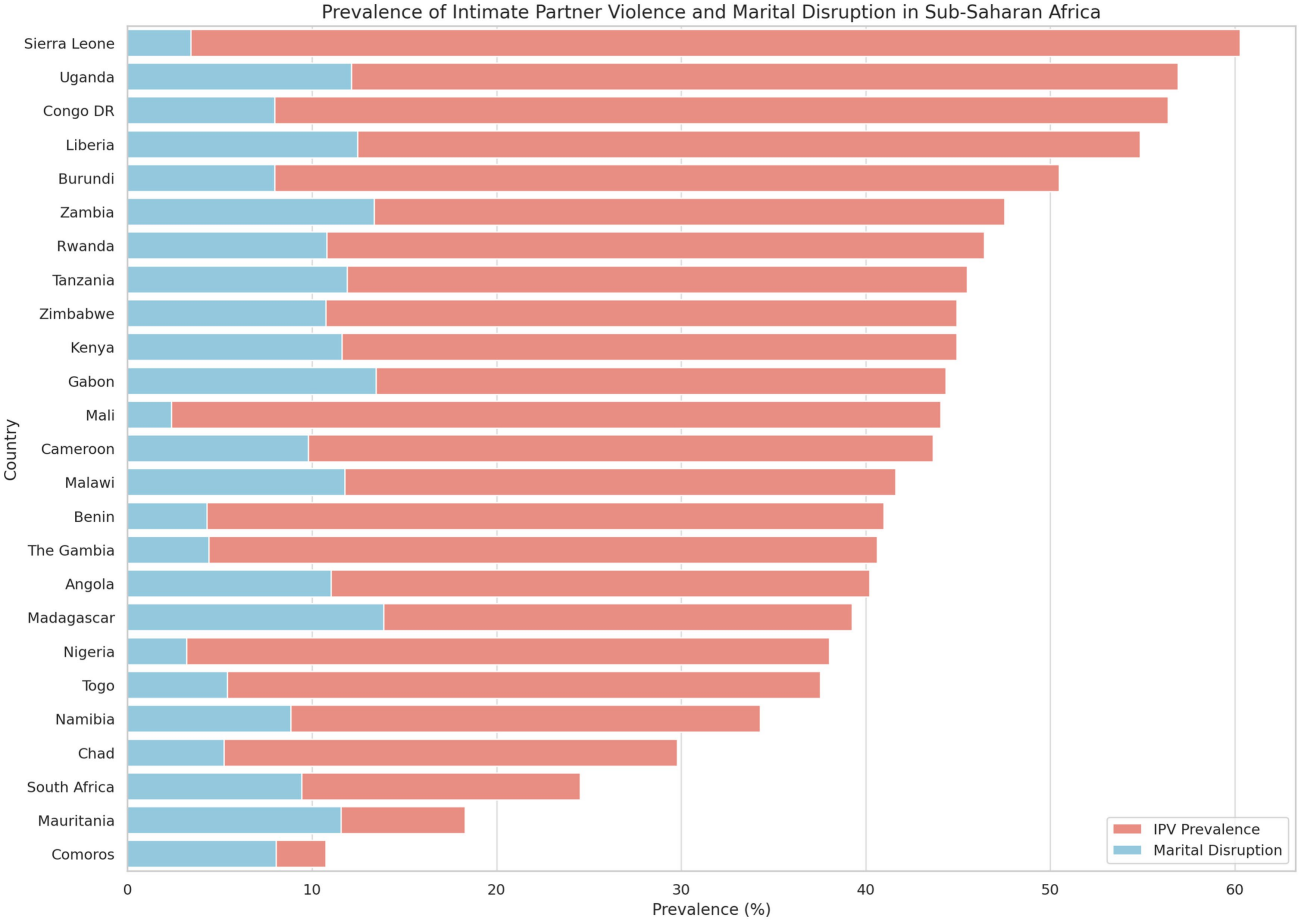
Prevalence of IPV and marital disruption in SSA. Sociodemographic characteristics of respondents by IPV.

Table 2 and Figure 1 present the prevalence of IPV and marital disruption among ever-married women across 25 countries in sub-Saharan Africa. Overall, IPV remains a pervasive issue in the region, with 43.23% of women reporting having experienced at least one form of IPV—whether emotional, physical, or sexual. This finding underscores the widespread nature of partner violence and its significant implications for women’s wellbeing, family stability, and public health.

**Table 2:** Prevalence of IPV and marital disruption in SSA.

Countries	IPV prevalence	Prevalence of marital disruption
Angola	3,005 (40.22%)	825 (11.04%)
Benin	1792 (40.98%)	188 (4.30%)
Burundi	3,511 (50.48%)	554 (7.97%)
Cameroon	1963 (43.66%)	441 (9.81%)
Chad	1,093 (29.80%)	192 (5.23%)
Comoros	269 (10.76%)	201 (8.04%)
Congo DR	3,130 (56.39%)	443 (7.98%)
Gabon	1,231 (44.34%)	374 (13.47%)
The Gambia	775 (40.62%)	84 (4.40%)
Kenya	5,534 (44.93%)	1,432 (11.63%)
Liberia	1,246 (54.87%)	283 (12.46%)
Madagascar	2,277 (39.25%)	805 (13.89%)
Malawi	2,181 (41.63%)	617 (11.78%)
Mali	1,458 (44.06%)	79 (2.39%)
Mauritania	595 (18.29%)	376 (11.56%)
Namibia	477 (34.27%)	123 (8.84%)
Nigeria	3,281 (38.04%)	277 (3.21%)
Rwanda	867 (46.41%)	202 (10.81%)
Sierra Leone	2,382 (60.27%)	136 (3.44%)
South Africa	546 (24.54%)	210 (9.44%)
Tanzania	3,346 (45.49%)	876 (11.91%)
Togo	1938 (37.53%)	279 (5.40%)
Uganda	4,143 (56.92%)	883 (12.13%)
Zambia	3,363 (47.53%)	945 (13.36%)
Zimbabwe	2,476 (44.94%)	593 (10.76%)
Total	52,879 (43.23%)	11,418 (9.33%)

The data reveal considerable variation across countries. The highest prevalence of IPV was recorded in Sierra Leone, where more than 6 in 10 women (60.27%) reported experiencing IPV. Other countries with particularly high prevalence rates include Uganda (56.92%), Congo DR (56.39%), and Liberia (54.87%). These figures suggest systemic societal, cultural, or institutional factors that may perpetuate IPV in these settings.

In contrast, Comoros reported the lowest IPV prevalence at 10.76%, followed by Mauritania (18.29%), and South Africa (24.54%). While these figures appear relatively low compared to the aforementioned countries, they still highlight a significant proportion of women facing violence within intimate relationships.

When examining marital disruption, defined as separation or divorce, a different pattern emerges. The overall prevalence of marital disruption was 9.33%, with Madagascar (13.89%), Zambia (13.36%), and Gabon (13.47%) reporting the highest rates. Interestingly, Mali, despite having a moderately high IPV rate (44.06%), reported the lowest level of marital disruption (2.39%). This suggests a cultural or normative influence where women may remain in violent unions due to social expectations, stigma, or lack of alternatives.

The divergent trends between IPV prevalence and marital disruption in some countries (e.g., high IPV but low disruption in Mali or Sierra Leone) point to the complex and context-specific nature of these experiences. Factors such as patriarchal norms, religious values, economic dependence, and societal attitudes toward divorce likely shape women’s responses to IPV.

These variations across countries highlight the importance of tailoring policy and intervention strategies to specific cultural and national contexts. Understanding where IPV is most prevalent—and where it is less likely to lead to marital disruption—can help inform targeted programs aimed at prevention, protection, and empowerment.

Table 3 below presents the distribution of ever-married women aged 15–49 across various sociodemographic characteristics and the prevalence of IPV within each category. The findings highlight important trends and disparities in the experience of IPV among subgroups in Sub-Saharan Africa. IPV prevalence is highest among women aged 25–29 years (22.56%), closely followed by those aged 30–34 (20.35%) and 20–24 (17.03%), suggesting that women in this age range were particularly vulnerable. Prevalence declines steadily among older age groups.

**Table 3:** Percentage distribution of sociodemographic characteristics of respondents by IPV.

Variable	Weighted *N*	Weighted %	IPV (%)	*p*-value
Age group				<0.001
15–19	6,956	5.68	4.79	
20–24	21,231	17.33	17.03	
25–29	27,618	22.55	22.56	
30–34	24,440	19.95	20.35	
35–39	19,348	15.80	16.16	
40–44	13,392	10.93	11.08	
45–49	9,492	7.75	8.04	
Residence				<0.001
Urban	45,755	37.36	33.0	
Rural	76,722	62.64	67.0	
Educational attainment				<0.001
No education	33,556	27.40	28.16	
Primary	46,292	37.80	41.53	
Secondary	35,418	28.92	26.83	
Tertiary	7,211	5.89	3.48	
Media exposure				<0.001
Not exposed	104,616	85.42	88.96	
Exposed	17,861	14.58	11.04	
Wealth index				<0.001
Poorest	24,282	19.83	24.00	
Poorer	24,400	19.92	21.38	
Middle	24,370	19.90	20.70	
Richer	25,297	20.65	19.22	
Richest	24,128	19.70	14.70	
Parity				<0.001
None	7,366	6.01	4.22	
1–4	79,681	65.06	63.17	
5+	35,430	28.93	32.61	
Number of co-wives				<0.001
None	103,035	84.13	82.21	
More than one	19,442	15.87	17.79	
Age of first cohabitation/relationship				<0.001
Less than 18	49,997	40.82	43.78	
18 years and over	72,480	59.18	56.22	
Duration of relationship				<0.001
0–4	28,439	23.22	19.15	
5–9	28,520	23.29	23.61	
10–14	23,988	19.59	20.72	
15–19	18,590	15.18	16.34	
20+	22,940	18.73	20.18	
Partner’s educational attainment				<0.001
No education	34,920	28.51	31.59	
Primary	39,481	32.24	33.73	
Secondary	37,339	30.49	28.97	
Tertiary	10,737	8.77	5.71	
Employment status				<0.001
Unemployed	33,510	27.36	22.97	
Employed	88,967	72.64	77.03	
Age difference				<0.001
Wife older	5,176	4.23	4.32	
0–5 years	45,969	37.53	36.55	
6 years and over	51,406	41.97	39.97	
Husband age unknown	19,926	16.27	19.15	
Empowerment				0 < 0.001
Not Empowered	27,899	22.78	20.60	
Empowered	94,578	77.22	79.40	
Partners controlling behavior				<0.001
Controlling	86,484	70.61	87.67	
Not controlling	35,993	29.39	12.33	
Alcohol consumption				<0.001
No	77,664	63.41	49.44	
Yes	44,813	36.59	50.56	
Drunkenness frequency				<0.001
Never	6,236	13.92	8.87	
Often	12,499	27.89	36.38	
Sometimes	26,078	58.19	54.75	
Father beats respondent’s mother				<0.001
No	92,255	75.32	65.22	
Yes	30,222	24.68	34.78	

Rural women reported a significantly higher experiences of IPV (67.0%) compared to their urban counterparts (33.0%), pointing to geographic disparities possibly shaped by access to services, education, and sociocultural norms. With respect to education, IPV was most common among women with primary education (41.53%), followed by those with no education (28.16%). The rate declined substantially among women with secondary (26.83%) and tertiary education (3.48%), reinforcing the protective effect of higher education. Media exposure is often considered a pathway to empowerment; the data show that 88.96% of IPV cases were among women not exposed to media, with a statistical significance.

Economic status appears to have a gradient effect: IPV was most prevalent among women in the poorest quintile (24.00%), and decreased progressively to 14.70% among the richest quintile. This confirms a strong inverse relationship between wealth and IPV. Parity also shaped IPV risk, with women having 1–4 children (63.17%) being most affected, followed by those with 5+ children (32.61%), while women with no children reported the lowest prevalence (4.22%).

Women in monogamous relationships (i.e., with no co-wives) reported slightly lower IPV prevalence (82.21%) compared to those in polygynous unions (17.79%). Surprisingly, women who began cohabiting at age 18 or older reported higher IPV prevalence (56.22%) than those who began earlier (43.78%), countering assumptions that early marriage increases IPV risk and suggesting complex relationship dynamics that warrant further investigation.

In terms of union duration, IPV peaked among women in relationships lasting 5–9 years (23.61%), followed by 10–14 years (20.72%). This indicates that IPV may intensify or persist in mid-duration unions.

Women whose partners had primary education (33.73%) or no education (31.59%) were most affected by IPV, whereas those with partners who attained tertiary education (5.71%) reported the lowest rates, underlining the positive influence of male partner education. Employment status is strongly associated with IPV: 77.03% of women who experienced IPV were employed, compared to 22.97% who were unemployed, suggesting economic participation does not necessarily confer protection and may even trigger tension in patriarchal settings.

IPV prevalence was also higher among women whose partners were more than 5 years older (39.97%), compared to those with smaller age gaps (36.55%) or younger husbands (4.32%). Women unaware of their partner’s age also had relatively high IPV levels (19.15%). Empowerment showed a mixed result: a greater proportion of IPV cases (79.40%) occurred among women categorized as empowered, potentially reflecting greater willingness to disclose abuse or heightened conflict due to shifts in traditional power dynamics.

Partner’s controlling behavior had a strong and consistent association with IPV. An overwhelming 87.67% of IPV cases were reported by women whose partners exhibited controlling behaviors, compared to only 12.33% among those whose partners were not controlling. Partner alcohol use and frequency of drunkenness were also important predictors. More than half of IPV victims (50.56%) had partners who drank alcohol. IPV was particularly common among those whose partners got drunk often (36.38%) or sometimes (54.75%), compared to just 8.87% among women whose partners never got drunk.

Finally, women who had witnessed their fathers beating their mothers reported higher IPV exposure (34.78%) than those who had not (65.22%), affirming the intergenerational transmission of violence and the influence of learned behaviors.

### Sociodemographic characteristics of respondents by marital disruption

Table 4 presents the distribution of ever-married women aged 15–49 by marital disruption status and various sociodemographic characteristics. The results reveal statistically significant associations between all the explanatory variables and marital disruption (*p* < 0.001).

**Table 4:** Percentage distribution of sociodemographic characteristics of respondents by marital disruption.

Variable	Weighted *N*	Weighted %	Marriage disruption (%)	*p*-value
Age group				<0.001
15–19	6,956	5.68	3.96	
20–24	21,231	17.33	14.64	
25–29	27,618	22.55	19.15	
30–34	24,440	19.95	19.83	
35–39	19,348	15.80	16.82	
40–44	13,392	10.93	14.64	
45–49	9,492	7.75	10.97	
Residence				<0.001
Urban	45,755	37.36	42.92	
Rural	76,722	62.64	57.08	
Educational attainment				<0.001
No education	33,556	27.40	20.83	
Primary	46,292	37.80	43.89	
Secondary	35,418	28.92	30.36	
Tertiary	7,211	5.89	4.93	
Media exposure				<0.001
Not exposed	104,616	85.42	85.30	
Exposed	17,861	14.58	14.70	
Wealth index				<0.001
Poorest	24,282	19.83	23.44	
Poorer	24,400	19.92	19.17	
Middle	24,370	19.90	19.77	
Richer	25,297	20.65	20.48	
Richest	24,128	19.70	17.15	
Parity				<0.001
None	7,366	6.01	5.34	
1–4	79,681	65.06	71.98	
5+	35,430	28.93	22.68	
Age of first cohabitation/relationship				<0.001
Less than 18	49,997	40.82	39.79	
18 years and over	72,480	59.18	60.21	
Duration of relationship				<0.001
0–4	28,439	23.22	17.15	
5–9	28,520	23.29	22.41	
10–14	23,988	19.59	19.33	
15–19	18,590	15.18	17.22	
20+	22,940	18.73	23.89	
Partner’s educational attainment				<0.001
No education	34,920	28.51	92.33	
Primary	39,481	32.24	2.35	
Secondary	37,339	30.49	4.31	
Tertiary	10,737	8.77	1.01	
Employment status				<0.001
Unemployed	33,510	27.36	20.20	
Employed	88,967	72.64	79.80	
Empowerment				<0.001
Not empowered	27,899	22.78	22.55	
Empowered	94,578	77.22	77.45	
Partners controlling behavior				<0.001
Controlling	86,484	70.61	81.16	
Not controlling	35,993	29.39	18.84	
Alcohol consumption				<0.001
No	77,664	63.41	50.28	
Yes	44,813	36.59	49.72	
Drunkenness frequency				<0.001
Never	6,236	13.92	6.11	
Often	12,499	27.89	48.42	
Sometimes	26,078	58.19	45.47	
Father beats respondent’s mother				<0.001
No	92,255	75.32	73.12	
Yes	30,222	24.68	26.88	

Age was a significant predictor of marital disruption. Marital disruption was most commonly reported among women aged 30–34 years (19.83%) and 25–29 years (19.15%), followed closely by those aged 35–39 years (16.82%) and 40–44 years (14.64%), indicating that disruption was most likely to occur during mid-marital years when relationships are more established.

In terms of residence, more than half of the women with disrupted marriages reside in rural areas (57.08%), although a sizable proportion (42.92%) live in urban settings, suggesting that marital disruption affects women across geographic locations.

Educational attainment showed a non-linear relationship with marital disruption. Women with primary education experience the highest rates of disruption (43.89%), followed by those with secondary education (30.36%). Interestingly, women with no formal education report lower disruption (20.83%) compared to the primary and secondary groups, while those with tertiary education report the lowest disruption (4.93%). These findings suggest that while higher education may have a protective role, the relatively lower disruption among women with no education could reflect other contextual factors, such as stronger adherence to traditional marital norms or limited agency to exit unions despite IPV.

Similarly, women who lacked media exposure reported disproportionately high levels of marital disruption (85.30%), though their larger population share may partly explain this. Nevertheless, this underscores the potential role of information access in marital stability.

Economic status shows a modest, but non-linear, association with marital disruption. Women in the poorest households reported the highest disruption (23.44%), followed by those in the richer group (20.48%). The lowest disruption was observed among women in the richest households (17.15%). These results suggest that while economic advantage may offer some protection against marital instability, the relationship is not strictly linear across all wealth quintiles.

Parity showed a clear concentration of marital disruption among women with 1–4 children (71.98%), compared to 22.68% among those with five or more children and 5.34% among women with no children. This suggests that marital instability is most pronounced during the early to mid-reproductive years, while lower rates among childless women may reflect fewer unions or shorter union durations, and lower rates among women with 5+ children may indicate greater marital stability in larger, long-established families.

Age at first cohabitation also correlates with marital disruption: 60.21% of disrupted unions involved women who began cohabiting or entered relationships at age 18 or older, indicating that delayed union formation does not necessarily guarantee marital stability.

Disruption is more common among women in relationships lasting 20 years or more (23.89%), followed by 5–9 years (22.41%), and 10–14 years (19.33%). This shows that disruptions can occur both early and late in the marital lifecycle.

An unusual trend appears in relation to partner’s education. An overwhelming 92.33% of disrupted marriages involve women whose partners had no formal education, whereas those with partners who had primary (2.35%), secondary (4.31%), or tertiary (1.01%) education were far less likely to report marital disruption, underlining the importance of male education in marital stability.

Marital disruption was more commonly reported among employed women (79.80%), compared to unemployed women (20.20%). Similarly, a greater proportion of disrupted unions occurred among women who were empowered (77.45%), possibly reflecting women’s agency to exit abusive or unsatisfactory marriages.

The relationship between partner’s behavior and marital disruption is also pronounced. A striking 81.16% of women in disrupted unions reported that their partners were controlling, suggesting emotional or psychological abuse as a factor contributing to separation.

In terms of alcohol consumption, just over half of the women with disrupted marriages reported that their partners did not drink (50.28%), while the remaining 49.72% had partners who did. However, among those with partners who drank, nearly half (48.42%) stated that their partners often got drunk, and another 45.47% said they sometimes got drunk, suggesting that alcohol abuse plays a substantial role in marital instability.

Finally, women who never witnessed their fathers beating their mothers reported higher levels of marital disruption (73.12%) than those who did (26.88%), contrary to expectations. This might suggest that those with no prior exposure to violence are less tolerant of dysfunctional relationships and more likely to exit.

In conclusion, Table 4 demonstrates that marital disruption among women is associated with a complex interplay of age, education, employment, empowerment, partner’s characteristics, and exposure to violence.

### Association between marital disruption and IPV

Table 5 presents the association between marital disruption and various forms of IPV, including physical, emotional, and sexual violence. The analysis reveals a statistically significant relationship between marital disruption and all forms of IPV (*p* < 0.001).

**Table 5:** Association between marital disruption and IPV.

Forms of IPV	Marital disruption		*p*-value
	No (%)	Yes (%)	
Physical violence	32,166 (85.55%)	5,434 (14.45%)	<0.001
Emotional violence	32,328 (84.89%)	5,753 (15.11%)	<0.001
Sexual violence	12,539 (83.17%)	2,537 (16.83%)	<0.001
Intimate partners violence	45,849 (86.71%)	7,030 (13.29%)	<0.001

Among women who reported physical violence, 14.45% experienced marital disruption. Similarly, 15.11% of women who experienced emotional violence and 16.83% of those subjected to sexual violence reported that their marriages had been disrupted. Although these percentages appear modest, they indicate that women who experience violence—regardless of the form—are more likely to have disrupted marriages than those who do not.

Overall, 13.29% of women who experienced any form of IPV reported marital disruption, while 86.71% remained in their unions despite experiencing violence.

These findings underscore the complex role IPV plays in marital dynamics. While not all abuse results in marital disruption, the experience of violence has the likelihood for marital disruption, especially in cases involving sexual violence.

### Logistic regression of sociodemographic correlates of IPV

Table 6 presents the results of the logistic regression analysis examining sociodemographic and relational correlates of experiencing any form of IPV among ever-married women in sub-Saharan Africa.

**Table 6:** Logistic regression of sociodemographic and relational correlates of IPV.

Variable	Category	Adjusted odds ratio (95% CI)
Age group	15–19	RC
	20–24	1.06 [0.99–1.13]
	25–29	0.94 [0.88–1.01]
	30–34	0.81 [0.75–0.88]*
	35–39	0.73 [0.66–0.80]*
	40–44	0.66 [0.59–0.72]*
	45–49	0.65 [0.59–0.73]*
Education	No education	RC
	Primary	1.16 [1.12–1.20]*
	Secondary	1.09 [1.05–1.14]*
	Tertiary	0.88 [0.81–0.95]*
Residence	Urban	RC
	Rural	0.95 [0.92–0.98]*
Media exposure	Not exposed	RC
	Exposed	0.93 [0.82–0.97]*
Wealth index	Poorest	RC
	Poorer	0.98 [0.95–1.02]
	Middle	*0.96 [0.92–1.00]*
	Richer	*0.96 [0.92–1.00]*
	Richest	0.86 [0.82–0.91]*
Parity	None	RC
	1–4	1.37 [1.29–1.45]*
	5+	1.52 [1.42–1.62]*
Number of co-wives	None	RC
	One or more	1.25 [1.21–1.30]*
Age at first cohabitation	<18 years	RC
	18 years and above	0.98 [0.95–1.01]
Duration of relationship (years)	0–4	RC
	5–9	1.31 [1.25–1.37]*
	10–14	1.50 [1.42–1.58]*
	15–19	1.61 [1.51–1.72]*
	20+	1.67 [1.54–1.81]*
Partner’s education	No education	RC
	Primary	0.91 [0.88–0.95]*
	Secondary	0.86 [0.83–0.90]*
	Tertiary	0.69 [0.64–0.73]*
Occupation	Unemployed	RC
	Employed	1.35 [1.31–1.40]*
Age difference (partner–wife)	Wife older	RC
	0–5 years	1.00 [0.94–1.06]
	≥6 years	0.93 [0.87–0.99]*
	Husband’s age unknown	1.42 [1.33–1.53]*
Empowerment	Not empowered	RC
	Empowered	1.03 [1.00–1.06]
Partner’s controlling behavior	Not controlling	RC
	Controlling	3.98 [3.84–4.11]*
Partner drinks alcohol	No	RC
	Yes	1.56 [1.52–1.61]*
Witnessed parental violence	No	RC
	Yes	2.21 [2.15–2.28]*
Constant	—	0.09 [0.08–0.11]

Reference category, RC. *Statistically significant values indicated by *p* < 0.05.

Compared to women aged 15–19, those in the 20–24 age group were slightly more likely to experience IPV (AOR = 1.06, 95% CI: 0.99–1.13), although this was not statistically significant. The odds of experiencing IPV consistently declined with age and were significantly lower among women aged 30–49, with the lowest likelihood observed among those aged 45–49 years (AOR = 0.65, 95% CI: 0.59–0.73).

Educational attainment showed a complex relationship with IPV. Women with primary or secondary education had higher odds of experiencing IPV compared to those with no education. However, those with tertiary education were significantly less likely to experience IPV (AOR = 0.88, 95% CI: 0.81–0.95).

Women residing in rural areas were slightly less likely to report IPV than urban dwellers (AOR = 0.95, 95% CI: 0.92–0.98). Similarly, media exposure was associated with reduced odds of IPV (AOR = 0.93, 95% CI: 0.82–0.97). Household wealth also showed a protective trend: women in the richest quintile were significantly less likely to report IPV (AOR = 0.86, 95% CI: 0.82–0.91) compared to the poorest group.

Parity was positively associated with IPV. Women who had given birth to 1–4 or 5+ children were more likely to experience IPV (AORs = 1.37 and 1.52, respectively) compared to those with no children. Having one or more co-wives also significantly increased the odds of IPV (AOR = 1.25, 95% CI: 1.21–1.30).

Marital history and partner characteristics played a crucial role. Women who began cohabiting at age 18 or older were slightly less likely to experience IPV, although this was not statistically significant. Longer relationship duration was associated with increased IPV risk, rising steadily from those in unions for 5–9 years (AOR = 1.31) to 20+ years (AOR = 1.67).

Partner’s education was protective: the likelihood of IPV decreased progressively with higher partner education, with women whose partners had tertiary education being the least likely to experience IPV (AOR = 0.69, 95% CI: 0.64–0.73).

Women who were employed were more likely to experience IPV than those who were not (AOR = 1.35, 95% CI: 1.31–1.40). A narrower or larger age difference with a spouse did not significantly affect IPV risk, except when the husband’s age was unknown (AOR = 1.42, 95% CI: 1.33–1.53).

Empowerment (defined by asset ownership or income generation) showed a marginal and non-significant increase in IPV odds (AOR = 1.03, 95% CI: 1.00–1.06).

Partner behavior variables revealed the strongest associations. Women whose partners exhibited controlling behavior were nearly four times more likely to experience IPV (AOR = 3.98, 95% CI: 3.84–4.11). Alcohol consumption by the partner nearly doubled the likelihood of IPV (AOR = 1.56, 95% CI: 1.52–1.61). Lastly, women who had witnessed their father beating their mother were more than twice as likely to experience IPV (AOR = 2.21, 95% CI: 2.15–2.28), highlighting the intergenerational transmission of violence.

### IPV and marital disruption

Table 7 presents the logistic regression results assessing the association between IPV and marital disruption. Women currently married or cohabiting was coded as “1” and “0” for those divorced or separated. The results show that IPV significantly reduced the likelihood of being in a current union. Women who reported experiencing any form of IPV had 56% lower odds of remaining married or cohabiting compared to women who did not report IPV (OR = 0.44, 95% CI: 0.42–0.46, *p* < 0.001).

**Table 7:** Logistic regression model of IPV and marital disruption.

Marriage	Odd ratio	Std. Err	*z*	*p*-value	[95% CI]
IPV (No)	RC				
IPV (Yes)	0.44	0.01	−40.70	<0.001	[0.42–0.46]
Constant	14.83	0.23	172.89	<0.001	[14.38–15.29]

This finding indicates that IPV is strongly associated with marital disruption, reinforcing the assertion that violence within relationships contributes to union breakdown. This is because IPV undermines trust, causes physical and psychological harm, and may ultimately push women toward separation or divorce. In other words, the analysis suggests that IPV is strongly associated with marital dissolution in sub-Saharan Africa, on the ground that experiences of violence within intimate partnerships substantially increase the risk of marital instability.

## Discussion

This study investigated the predictors of IPV and associated marital disruption among ever-married women in sub-Saharan Africa, drawing on multi-country Demographic and Health Survey data. The findings revealed a high overall prevalence of IPV at 43.23%, with substantial variation across countries—from 10.76% in Comoros to more than 60% in Sierra Leone. This underscores the pervasive yet context-specific nature of IPV in the region and signals the urgent need for tailored interventions.

Several predictors of IPV emerged from the analysis. Women with lower levels of education were disproportionately affected, especially by sexual IPV, consistent with findings from Ethiopia (Abeya et al., 2011), India (Ackerson et al., 2008), South Africa (Abrahams et al., 2013), and the WHO multi-country study (World Health Organization, 2012). Educational attainment enhances women’s resources, awareness, and social capital, serving as a protective factor. Childhood exposure to parental violence also significantly predicted IPV, confirming global evidence that intergenerational cycles of violence perpetuate abuse (Kalamar et al., 2018; Devries et al., 2017; Wandera et al., 2015). Partner controlling behaviors strongly predicted IPV across all forms (Wandera et al., 2015; World Health Organization, 2012), reflecting entrenched patriarchal hierarchies that normalize male dominance. Other predictors included partner alcohol consumption and intoxication (Mulawa et al., 2018; Tumwesigye et al., 2012; Heise, 2011), younger age at marriage, relationship duration, lower socioeconomic status (Osinde et al., 2011; Jeyaseelan et al., 2007; Vyas and Watts, 2008), and higher parity (Babu and Kar, 2010; Hindin et al., 2008). Together, these findings highlight the multifaceted and structural drivers of IPV in the region.

The study also demonstrated a strong and statistically significant association between IPV and marital disruption. Logistic regression analysis revealed that women who experienced any form of IPV had 56% lower odds of remaining in a marital or cohabiting union compared to women not reporting IPV. Among the forms of abuse, sexual violence showed the strongest association with marital breakdown, suggesting it may be the least tolerated within conjugal relationships. These results resonate with Seidu et al. (2021), who found IPV to be a predictor of union dissolution in Ghana, and with evidence from Uganda (Wagman et al., 2016), Spain (Ferrer-Perez et al., 2020), and multi-country analyses (Stöckl et al., 2014), which consistently confirm the destabilizing effect of IPV on intimate unions.

The consequences of IPV extend beyond individual relationships to broader psychosocial and structural domains. IPV erodes trust, respect, and emotional bonds, undermining relationship sustainability and exposing women to physical and psychological harm (Capaldi et al., 2012; Rountree and Mulder, 2017). Structural barriers—including stigma, limited economic independence, and weak legal protections—often compel women to remain in abusive unions despite severe risks (Decker et al., 2015; Gibbs et al., 2018). Yet, women with greater empowerment, educational attainment, or media exposure may have both the resources and agency to leave abusive relationships, demonstrating the dual role of empowerment in heightening awareness while also provoking backlash in patriarchal contexts.

These findings carry critical implications for policy and intervention priorities. To reduce IPV and its destabilizing impact on marriages, strategies must address both individual-level predictors and structural determinants. This includes expanding women’s access to education and economic opportunities, engaging men and communities to challenge patriarchal norms, curbing harmful behaviors such as alcohol abuse, and strengthening legal protections for women experiencing IPV. Culturally sensitive empowerment programs are essential, balancing the promotion of women’s autonomy with efforts to transform unequal gender norms that underpin violence. Future research should adopt longitudinal designs to explore causal pathways between IPV predictors and marital disruption and investigate how contextual differences mediate these dynamics.

Summarily, this study contributes robust multi-country evidence that IPV in sub-Saharan Africa is driven by identifiable predictors and strongly associated with marital disruption. Addressing IPV requires comprehensive and context-specific policies that not only protect women’s rights and health but also promote marital stability and social cohesion- outcomes that are vital for sustainable development in the region (World Health Organization, 2013a, 2013b).

### Contribution to the broader discourse on gender-based violence and the SDGs

The findings of this study significantly contribute to the broader discourse on gender-based violence (GBV) by empirically illustrating how IPV is not only widespread but also deeply consequential for marital stability among women in sub-Saharan Africa. This aligns with and reinforces the global agenda set by the United Nations Sustainable Development Goals (SDGs), particularly Goal 5: Achieve gender equality and empower all women and girls. Target 5.2 specifically aims to eliminate all forms of violence against women and girls in both public and private spheres, including IPV. By uncovering the statistically significant links between IPV and marital disruption, this study provides compelling evidence that IPV is not merely a private issue but a structural barrier to women’s well-being, autonomy, and social stability.

Furthermore, the study’s identification of key risk factors—such as low education, alcohol abuse, and controlling partner behavior—underscores the need for intersectional and context-sensitive interventions. These insights are critical for informing integrated policy responses that address multiple SDGs simultaneously, including Goal 3 (Good Health and Well-being), Goal 4 (Quality Education), and Goal 10 (Reduced Inequalities). By illuminating the pathways through which IPV disrupts women’s lives and relationships, the study calls for a multi-sectoral approach that empowers women economically, enhances access to education and legal protection, and transforms harmful gender norms. In doing so, it contributes to building a more inclusive and equitable society, where women are safe, supported, and free to thrive.

## Conclusion and recommendations

This study provides critical insights into the predictors of IPV and associated marital disruption among ever-married women in sub-Saharan Africa. The analysis revealed that IPV remains highly prevalent across the region, affecting over four in ten women, and that its occurrence is strongly linked with marital breakdown. Women who reported experiencing IPV were significantly less likely to remain in marital or cohabiting unions, with sexual violence emerging as the form most strongly associated with disruption. These findings demonstrate that IPV is not only a violation of women’s rights and well-being but also a destabilizing force within families, with long-term social and economic consequences.

The study identified a number of predictors that shape IPV risk and marital disruption. Women with lower levels of education, those from poorer households, and those who witnessed parental violence during childhood were disproportionately affected. Partner characteristics, particularly alcohol consumption, intoxication, and controlling behaviors, further heightened the risk of IPV. In contrast, higher educational attainment and media exposure served as protective factors, reducing both IPV and the likelihood of union instability. Relationship characteristics such as longer duration of marriage and higher parity also emerged as significant, pointing to the complex social and cultural pressures that keep women in abusive unions despite ongoing harm. While empowerment is often theorized as a protective factor against IPV and marital disruption, results showed only a marginal, non-significant effect, with empowered women having slightly higher odds of disruption. This suggests that economic empowerment neither clearly protected against nor increased the risk of marital instability.

The findings underscore the urgent need for interventions that address both the individual and structural dimensions of IPV. Efforts to expand educational opportunities for women remain crucial, as education not only reduces vulnerability to abuse but also equips women with the resources and social capital necessary to make informed decisions about their relationships. Economic empowerment is equally important, as women with greater financial independence were less likely to experience IPV and more likely to exercise agency in leaving abusive marriages. Preventing intergenerational cycles of violence also requires early interventions that challenge the normalization of spousal abuse and promote healthier models of relationships for future generations.

At the same time, addressing partner-level predictors such as alcohol use and controlling behaviors is essential. Public health interventions targeting harmful drinking patterns, alongside community campaigns to challenge controlling and patriarchal attitudes, can help reduce IPV prevalence. Strengthening legal protections and ensuring effective enforcement of existing laws against IPV are also critical to support women experiencing IPV and provide them with safe avenues for redress. Embedding IPV screening and referral services into routine health care—particularly within maternal and reproductive health programs—can facilitate early detection and support for women at risk.

Overall, this study highlights that IPV, and marital disruption are deeply intertwined phenomena shaped by identifiable predictors. Effective responses must therefore combine structural interventions that empower women with relational and behavioral strategies that address partner risk factors. By integrating IPV prevention into national development agendas, promoting gender equality, and expanding support systems, sub-Saharan African governments and stakeholders can reduce IPV prevalence, protect women’s rights, and foster more stable and resilient families.

## Study limitations

While this study provides a robust estimation of the prevalence of different forms of IPV among ever-married women aged 15–49 and offers valuable insight into the relationship between IPV and marital disruption using nationally representative data, several limitations must be acknowledged.

First, the use of cross-sectional data limits the ability to draw causal inferences. Although associations can be identified, the temporal sequence between IPV and marital disruption cannot be definitively established. Longitudinal studies would be more appropriate for capturing the dynamics and directionality of this relationship over time.

Second, the reliance on self-reported data introduces the potential for reporting bias. IPV remains a highly sensitive topic, and cultural norms surrounding gender roles, marriage, and family privacy—particularly prevalent in many African societies—may contribute to social desirability bias and underreporting. Women may feel compelled to withhold disclosures of abuse due to stigma, fear of retaliation, or pressure to preserve family honour.

Moreover, the analysis was constrained by the availability of variables within the Demographic and Health Survey datasets. Several potentially relevant predictors established in prior research—such as provocation by partners, communication difficulties, mental health conditions, and stress—were not captured (Garcia-Moreno et al., 2006; World Health Organization, 2013a,b). The exclusion of these factors may have limited the explanatory power of the models and the ability to fully capture the complexity of IPV dynamics.

Despite these limitations, the study provides meaningful contributions to the understanding of IPV and its implications for marital stability in sub-Saharan Africa. Future research should incorporate longitudinal designs and a broader set of psychosocial and relational variables to deepen insights and inform more targeted interventions.

## Data Availability

The original contributions presented in the study are included in the article/supplementary material, further inquiries can be directed to the corresponding author.
